# Supplementary material for: Realization of wafer-scale nanogratings with sub-50 nm period through vacancy epitaxy
Source: Nat Commun. 2019 Jun 4;10:2437. doi: 10.1038/s41467-019-10095-2 (PMC6547753; doi:10.1038/s41467-019-10095-2)
Supplement: Supplementary file 1 — Supplementary Information [file 41467_2019_10095_MOESM1_ESM.pdf]

Supplementary Information

Realization of wafer-scale nanogratings with sub-50 nm period through vacancy epitaxy

Qiushi Huang[2,#], *Qi jia*[1,4,#], *Jiangtao Feng*[2], *Hao Huang*[1,7], *Xiaowei Yang*[2,7], *Joerg Grenzer*[3], *Kai Huang*[1,4], *Shibing Zhang*[1,4], *Jiajie Lin*[1,4], *Hongyan Zhou*[1,4], *Tangui You*[1], *Wenjie Yu*[1], *Stefan Facsko*[3], *Philippe Jonnard*[5], *Meiyi Wu*[5], *Angelo Giglia*[6], *Zhong Zhang*[2], *Zhi Liu*[1,7], *Zhanshan Wang*[2], *Xi Wang*[1,4] & *Xin Ou*[1,]*

[1]State Key Laboratory of Functional Materials for Informatics, Shanghai Institute of Microsystem and Information Technology, CAS, 20050 Shanghai, China.

[2]Key Laboratory of Advanced Micro-Structured Materials MOE, Institute of Precision Optical Engineering, School of Physics Science and Engineering, Tongji University, Shanghai 200092, China.

[3]Institute of Ion Beam Physics and Materials Research, Helmholtz-Zentrum Dresden-Rossendorf, Bautzner Landstrasse 400, 01328 Dresden, Germany.

[4]University of Chinese Academy of Sciences, Beijing 10049, China.

[5]Sorbonne Université, Faculté des Sciences et Ingénierie, UMR CNRS, Laboratoire de Chimie Physique - Matière et Rayonnement, boîte courrier 1140, 4 place Jussieu F-75252 Paris cedex 05, France.

[6]CNR Istituto Officina Materiali 34149 Trieste, Italy.

[7]School of Physical Science and Technology, ShanghaiTech University, Shanghai 201210, China.

# These authors contributed equally to this work.

*Corresponding author: ouxin@mail.sim.ac.cn

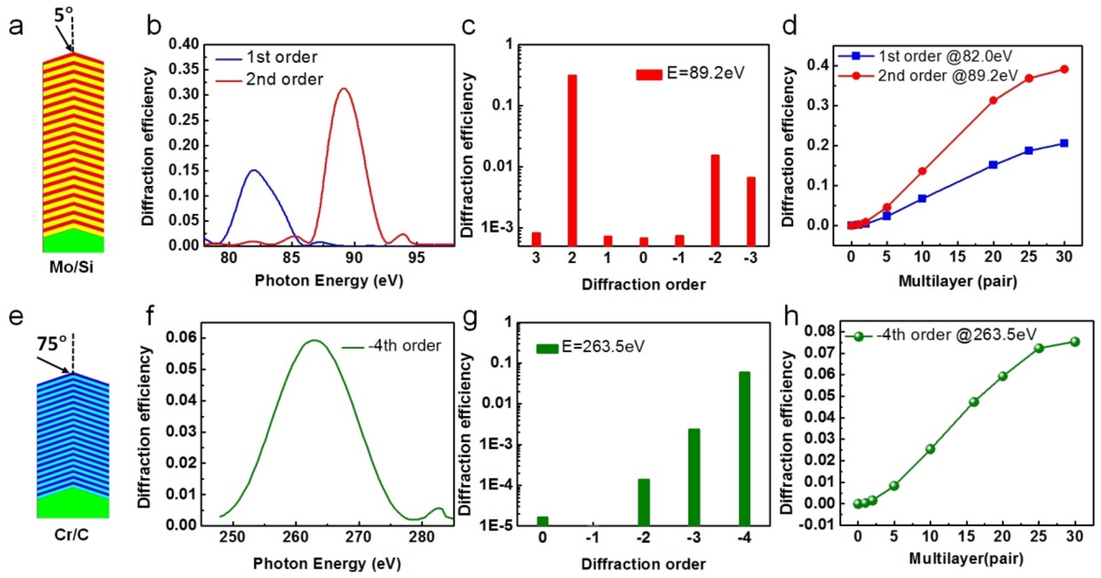

Supplementary Figure 1 Design and simulation of the multilayer nanogratings (a) Schematic of the Mo/Si MBG at an incident angle of 5°. (b) Simulated diffraction efficiency of the 1st (blue line) and 2nd (red line) order of the Mo/Si MBG versus the photon energy. (c) Simulated diffraction efficiency of the different orders of the Mo/Si MBG at an incident angle of 5° and photon energy of 89.2 eV. (d) Simulated efficiency of the 1st (blue line) and 2nd (red line) order of the Mo/Si MBG with increasing number of multilayer pairs (bilayers). (e) Schematic of the Cr/C MBG at an incident angle of 75°. (f) Simulated diffraction efficiency of the -4th order of the Cr/C MBG versus the photon energy. (g) Simulated efficiency of different orders of the Cr/C MBG at an incident angle of 75° and photon energy of 263.5 eV. (h) Simulated efficiency of the -4th order of the Cr/C MBG with increasing multilayer pairs.

Supplementary Table 1 Parameters of the Mo/Si multilayer nanograting used for simulation

Period (nm)	Blaze angle (°)	d_{Mo} (nm)	d_{Si} (nm)
50	19	3.25	4.88

Supplementary Table 2 Parameters of the Cr/C multilayer nanograting used for simulation

Period (nm)	Blaze angle (°)	d_{Cr} (nm)	d_{C} (nm)
60	19	1.92	2.88

Supplementary Note 1

The periodic sawtooth structures with a period of 46~78 nm were fabricated by self-assembly of vacancies. To demonstrate the potential application of these structures for EUV and soft X-ray gratings, we designed two multilayer nanogratings using Mo/Si and Cr/C multilayers, which can work around the photon energies of 85-95 eV or 265-275 eV, respectively. To achieve the resonant maximum efficiency, the d-spacing of the multilayer and the period and facet angle of the nanograting need to be matched to obey the grating equation and the generalized Bragg condition. The theoretical structural parameters are shown in Supplementary Table 1 and 2. The simulation was performed based on the coupled wave theory. For the Mo/Si multilayer grating, the incidence angle was 5°, as shown in Supplementary Figure 1a. The 2nd order is the resonant order according to the designed structure parameters, whose diffraction efficiency can reach as high as 32% at 89.2 eV with 20 bilayers (Supplementary Figure 1b-c). The dependence of the diffraction efficiency of the 1st and 2nd order with the number of bilayers was also investigated via simulations. The efficiency of the two orders will be both improved with more bilayers because of the higher reflectance (Supplementary Figure 1d). Nevertheless, to avoid the smoothing of the grating structure during the deposition of thick multilayers, 20 bilayers was selected for the first demonstration. The Cr/C MBG shows a similar tendency as the Mo/Si MBG (Supplementary Figure 1e-h). According to the designed structure parameters, the -4th order is the resonant order whose efficiency can reach 6% at a photon energy of 253.5 eV with an incident angle of 75° and 20 bilayers.
